# Associations between neighbourhood deprivation and engagement in arts, culture and heritage: evidence from two nationally-representative samples

**DOI:** 10.1186/s12889-021-11740-6

**Published:** 2021-09-16

**Authors:** Hei Wan Mak, Rory Coulter, Daisy Fancourt

**Affiliations:** 1 Department of Behavioural Science and Health, Institute of Epidemiology & Health Care, University College London, 1-19 Torrington Place, London, WC1E 7HB UK; 2 Department of Geography, University College London, Pearson Building, Gower Street, London, WC1E 6 BT UK

**Keywords:** Arts and cultural engagement, Neighbourhood deprivation, Propensity score matching, Geographical inequalities

## Abstract

**Background:**

Previous research has shown the benefits of arts and cultural engagement for physical, mental and social wellbeing. This engagement is socially and geographically patterned. Yet it remains unclear whether place-based attributes are associated with engagement behaviour independent of individual factors. Therefore, the aim of this cross-sectional study was to robustly disentangle associations between geographical deprivation and arts engagement from the individual socio-demographic factors that tend to correlate with residential locations.

**Methods:**

Two different samples drawn from two representative surveys of adults living in England were compared – Understanding Society Wave 2 (2010/12) (*N* = 14,782) and Taking Part survey (2010/11) (*N* = 4575). Propensity score matching (PSM) was applied to investigate the association between neighbourhood deprivation (20% most deprived vs 20% least deprived) and arts engagement (arts participation, cultural attendance and museums and heritage engagement).

**Results:**

Higher levels of neighbourhood deprivation were associated with lower arts, culture and heritage engagement independent of individuals’ demographic backgrounds, socio-economic characteristics and regional locations. When exploring subcategories of deprivation, similar results were obtained across deprivation domains. Results were also consistent when using more distinct categories of deprivation (i.e. 10% most deprived vs 10% least deprived) and when comparing people living in the 20% most deprived neighbourhoods with those living in the 40% medium-deprived areas.

**Conclusion:**

This study is the first to apply a robust PSM technique to examine the association between neighbourhood deprivation and arts engagement using two nationally-representative samples. Results show that neighbourhood deprivation may act as a barrier that could prevent people from engaging in the arts, which in turn may exacerbate social and health inequalities. This highlights the importance of place-based schemes that focus on increasing individual motivation and capacity to engage in arts and cultural activities, especially in areas of high deprivation.

**Supplementary Information:**

The online version contains supplementary material available at 10.1186/s12889-021-11740-6.

## Introduction

There is a growing body of literature suggesting that the arts can benefit physical, mental and social wellbeing [1]. Previous studies have shown that arts participation (e.g. playing music, dancing and doing textile crafts), cultural attendance (e.g. going to theatres) and visiting museums and heritage sites can all lead to positive psychological, physiological, social and behavioural responses. Such responses are, in turn, associated with the prevention, management and treatment of a range of different health outcomes. These include encouraging health-promoting behaviours [2, 3], supporting child development [4, 5], preventing mental illness [6, 7], enhancing social cohesion [8, 9] and reducing the risk of early mortality [10].

However, despite the considerable volume of evidence on the benefits of arts participation and cultural and heritage engagement (hereafter collectively termed “arts engagement”), access to and participation in these activities are rarely uniform. Research has suggested that individuals with higher levels of education and socio-economic position (SEP) are more likely to engage with the arts than their less advantaged peers [11–14]. Explanations for this include that people from more advantaged backgrounds tend to possess greater monetary resources, and can have greater cultural inclination and skills (often cultivated in childhood through parental encouragement) to participate in arts and cultural activities [15–17]. This is particularly relevant for cultural engagement which usually involves expenses such as the cost of entrance, ticket and programme fees.

Furthermore, arts engagement also appears to vary geographically. For example, levels of arts engagement in England have been found to be higher amongst those living in affluent countryside locales, wealthier areas, and cosmopolitan neighbourhoods [13, 14, 18, 19]. One possible explanation is that these geographical patterns may simply reflect underlying differences in the socio-economic characteristics of individuals living in these areas. On the other hand, it is also possible that individuals’ arts engagement is directly affected by spatial factors. Neighbourhood characteristics or physical structures (e.g. unsafe streets, poor transport links) may predispose more deprived communities to lower engagement in the arts due to a lower number or range of cultural assets (such as arts venues and groups within the area) and poor accessibility to the assets [18]. In addition to personal and area characteristics, various arts engagement behaviours across neighbourhoods may also be explained by area-specific social processes, including social contagion (e.g. behaviours, attitudes, role models in the arts may be influenced by peers living in the same neighbourhood), collective socialisation (e.g. individuals may be encouraged to engage in the arts if their neighbours have high levels of engagement) and social networks (e.g. individuals may become more aware of arts and cultural activities in local areas (particularly those with strong ties) through interpersonal communication) [20]. However, it is also plausible that arts engagement is viewed as an activity for individuals with high SEP, which may become a source of disamenity for those living in deprived areas who are typically less well-off [20]. On a related note, people living in deprived neighbourhoods may be more likely to experience psychological barriers such as a lack of confidence and a fear of not fitting in [21].

Given the health benefits of arts engagement, there is growing academic and policy interest in quantifying and reducing geographical disparities in arts engagement in Britain. For example, place-based funding streams such as the “Great Place Scheme” — an initiative originating with the Department for Digital, Culture, Media and Sport (DCMS)’s The Cultural White Paper in 2016 — was launched to fund local communities to embed culture in local authority’s plans and policies, support cultural offer and increase cultural engagement [22]. In addition, the “Creative People and Places” project funded by Arts Council England takes the stance that arts engagement can be increased through investing in places of high deprivation or low cultural opportunity [23]. Other schemes such as social prescribing, which connects individuals to cultural activities within their community through health and social care referrals [24–26], take a more individual-level approach to boosting arts engagement (alongside engagement in other community activities). But there are concerns that this may not work as effectively in areas of higher deprivation, where resources and services of arts and culture are more scarce and thus there may be lower uptake of the prescription and reduced benefits [27].

Whilst previous studies showed an association between arts behaviours and place independent of individual SEP [13, 14, 18, 28], the use of observational data and regression approaches may not fully deal with the problem of residual confounding caused by particular types of people selectively moving in and out of different sorts of places. Not accounting for these confounding issues could potentially risk over- or under-estimating the effect of neighbourhood on arts engagement. Further, many of the place-based schemes and interventions may only provide a one-off boost to engagement in the arts and it is unknown how place may shape the regular patterns of engagement that would have greater long-term benefits for health and wellbeing.

Therefore, in this study we sought to robustly disentangle associations between geographical deprivation and arts engagement from the individual measures of SEP that tend to correlate with residential locations. While it is not ethical or practical to address this question using randomised trials (i.e. by randomly assigning participants to deprived and non-deprived areas), this study uses propensity score matching (PSM) to effectively simulate experimental conditions from observational data [29, 30]. This is achieved by identifying “treatment” (people living in the most deprived neighbourhoods) and “control” groups (people living in the least deprived neighbourhoods), and matching individuals in the “treatment” group with one or more similar individuals in the “control” group such that the matched samples share very similar distributions on all observed covariates considered in the analysis. This approach helps remove the effects of identified confounders so that any remaining differences in the level of engagement between treatment and control groups can be more plausibly attributed to differences in neighbourhood deprivation (i.e. residence in deprived vs non-deprived areas).

We examined arts engagement amongst adults living in England cross-sectionally using data from Understanding Society: The UK Household Longitudinal Study (UKHLS) (2010/12). As a robustness check, we also analysed data from the Taking Part (TP) survey (2010/11) that serves as the main evidence source for DCMS to understand public engagement with the arts and cultural sector [31]. Both datasets share nearly identical measures of arts engagement and very similar measures of neighbourhood deprivation (the English Index of Multiple Deprivation, henceforth IMD) and are therefore useful for a cross-data comparison study.

This study was the first to examine whether engagement in arts and cultural activities was associated with area deprivation using the PSM technique on two nationally-representative samples.

## Materials and methods

*Sample 1 -* UKHLS is a nationally-representative survey that aims to interview over 50,000 individuals from around 30,000 households annually [32]. The survey contains a rich set of variables on arts and cultural participation, demographic background and socio-economic characteristics. We used Wave 2 data (2010–12; response rate = 84%) and focused on participants living in England (*N* = 38,069) and who provided full data across all measures (*N* = 36,472). We compared participants living in the 20% most deprived neighbourhoods (*N* = 7728) with those living in the 20% least deprived neighbourhoods (*N* = 7054) (total *N* = 14,782). Neighbourhoods were defined as 2011 census Lower Layer Super Output Areas (LSOAs). In the 2011 census there were 34,753 LSOAs in England and Wales (of which 32,844 LSOAs were in England) with populations ranging from 1000 to 3000 (mean = 1614) and household numbers ranging from 400 to 1200 (mean = 672) [33, 34]. The University of Essex Ethics Committee approved UKHLS and participants provided informed consent.

*Sample 2 –* TP is a nationally representative survey run by DCMS in order to inform the development and evaluation of their policy [35]. The survey has been approved by NatCen Social Research’s Research Ethics Committee and participants provided informed consent. TP collects data on engagement in wide-ranging activities including arts, museums and galleries, archives, libraries, heritage and sport, and provides data on geographical characteristics (which have already come attached to TP) including the IMD, regional locations and rural-urban classification. We used data from participants interviewed in 2010/11 (equivalent to UKHLS Wave 2) (*N* = 14,075; response rate = 57.3%). As with UKHLS, we only focused on respondents who provided complete response across all measures (*N* = 11,282) and compared participants who lived in the 20% most deprived neighbourhoods (*N* = 2261) to those who lived in the 20% least deprived neighbourhoods (*N* = 2314) (Total *N* = 4575).

### Measures

Level of neighbourhood deprivation was measured using the 2015 IMD in the UKHLS and 2007 IMD in TP. Both IMDs 2007 and 2015 used broadly the same methodology, domains and indicators to derive the decile index and, in general, most neighbourhoods do not experience major changes in their relative deprivation over short periods of time [36]. IMD is comprised of seven domains of deprivation that are each computed from a range of indicator variables compiled from various sources: income, employment, education, skills and training, health and disability, crime, barriers to housing and services, and living environment. These domains are then combined and weighted to generate a single score and rank position based on academic literature and statistical assessments [37].

Frequencies of arts engagement were measured through detailed questions asking respondents how often they had done particular activities, attended any cultural events or visited museums and heritage sites in the last 12 months (see Supplementary Table 1 for a list of activities).

Our PSM models incorporated possible confounding variables that might be associated with both living in a deprived area and/or frequency of arts engagement [38–40]. These included respondents’ age (aged under 35, aged 35–54, aged 55 or above), gender (female vs male), ethnicity (White, Asian/Asian British, Black/Black British, mixed/others), whether respondents were living alone, marital status (single and never married, married/in cohabitation, separated/divorced/widowed), whether respondents had children under 16 in the household, educational levels (university degree, advanced (higher degree/A-level), GCSE or equivalent, other/no qualification), occupational status (managerial/professional, intermediate/small employment/own account, lower supervision or technician/semi-routine or routine, not working (including unemployed, retired, full-time student, people with long-term health conditions)), annual personal gross income (under 20 k, 20-35 k, 35 k+), housing tenure (house owner, social renter, private renter), collapsed regional locations (North, Midlands, South), and settlement type (urban areas vs rural areas) [11–13, 19, 41].

### Statistical analysis

Our analysis used PSM to estimate the average treatment effect on the treated (ATT). The ATT measures the difference between the average outcome measure for respondents who were living in the *most* deprived neighbourhoods (the “treatment” group), and the average outcome measure for the sample group under the hypothetical scenario that they were living in the *least* deprived neighbourhoods (the “control” group). Compared to traditional regressions like OLS, the advantage of using PSM is that it can control for a large set of confounders, specifically individuals’ selection on observables into living in a deprived area, e.g. those who are in managerial/professional roles (and who are less likely to live in a deprived neighbourhood) are also the ones who are more likely to engage in the arts. Not taking into account such selection effects may bias the estimated effect of local deprivation. Further, PSM allows the treated and control individuals to be more comparable by matching their propensity scores, and hence to have similar “pre-treatment” characteristics (or covariates). Balancing these covariates can help reduce confounding issues and obtain a more unbiased estimate of the treatment effect. However, it is important to note that such comparability is conditioned to the observed and measurable covariates and may not hold for unmeasured ones [30]. Including all relevant observed covariates in a propensity score model is thus crucial.

In the analysis, we used unweighted PSM models and applied the kernel matching method with cross-validation bandwidth [42]. This matching method constructs the calculated propensity score to match treated cases to a weighted average of control cases. When the estimated propensity score of a control case is closer to the treated case, the control case receives a higher weight. More information is therefore taken from the matches whose propensity scores are closer to each other while those whose scores are distal are down-weighted [29, 43, 44]. Common support is imposed so that only observations within the overlap of the propensity score between treated and control units were considered as potential matches. To reduce bias due to residual differences after matching and to obtain an unbiased estimate of the treatment effect, regression adjustment was also applied on the matched sample (also known as a doubly robust approach) [42, 45, 46]. 95% confidence intervals were computed using bootstrapping techniques (clustering sampling design) with 100 replications. In our main analysis, 40% of the sample were analysed (i.e. 20% most deprived vs 20% least deprived neighbourhoods). Of the analytical sample, around 5.0% of cases in UKHLS (where 3.3% was due to missing data on the occupational status variable) and 17.8% of the TP sample were discarded due to missing data (where 16.6% was due to missing data on the income variable). Listwise deletion was used to remove cases with missing data. High quality of matching was also achieved. As shown in Supplementary figures s1a to s2b, the density distributions of the treatment and control groups overlap across two study samples, indicating good balances of the observed variables between the groups after matching. Further, the standardised mean differences of the covariates were very close to 0 after matching. These assessments suggest that the observed differences in demographic composition, socio-economic characteristics and regional locations between the treatment and control groups after matching are minimal, and that the possibility of residual confounding should have also been minimised.

To better understand which aspects of deprivation are most significantly associated with arts engagement, we used data from the UKHLS (which has a larger sample size and less missing data than TP) and repeated the analyses using the 7-subcategory of the IMD as the “treatment” variable (using 20% as the threshold – 20% most deprived vs 20% least deprived). In addition, the main analysis was repeated but using a 10% cut-off point for area deprivation (i.e. comparing 10% most deprived vs 10% least deprived neighbourhoods). Finally, we compared groups in the 20% most deprived neighbourhoods with groups in the 40% medium-deprived neighbourhoods (i.e. IMD decile ranks 4–7) to identify which levels of deprivation were associated with arts engagement. All methods were performed in accordance with the relevant guidelines and regulations. The analyses were implemented in Stata v16.1.

## Results

The unweighted demographic, socio-economic and geographical profiles of the two samples from the two different nationally representative data sources were similar, apart from the TP sample having fewer respondents from an ethnic minority background, more people living alone and with children under age 16, and more respondents who were not in employment (Table 1).

**Table 1:** Descriptive statistics of Understanding Society Wave 2 (2010/12) and the Taking Part survey (2010/11)

	Understanding Society Wave 2 (2010/12) (***N*** = 14,782)	Taking Part (2010/11) (***N*** = 4575)
	**Mean (SD) / %**	**Mean (SD) / %**
*Arts engagement frequency*		
Arts participation^1^	3.27 (2.11)	3.52 (2.10)
Cultural attendance^1^	2.00 (1.54)	2.15 (1.52)
Museums and heritage sites^1^	1.67 (1.53)	2.00 (1.51)
*Demographic backgrounds*		
Age		
Under 35	28.7%	26.6%
35–54	37.2%	36.2%
55 or above	34.1%	37.2%
Gender		
Female	55.9%	56.5%
Male	44.1%	43.5%
Ethnicity		
White	77.9%	87.9%
Asian/Asian British	13.1%	5.88%
Black/Black British	6.24%	4.28%
Mixed/other	2.83%	1.97%
Living alone		
Yes	14.4%	26.8%
No	85.6%	73.2%
Partnership status		
Single and never married	21.9%	25.5%
Married or in cohabitation	63.6%	51.9%
Separated or divorced or widowed	14.4%	22.6%
Presence of child (ren) under 16 in the household		
Yes	19.8%	31.7%
No	80.2%	68.4%
*Socio-economic position (SEP)*		
Educational levels		
University degree	32.5%	23.4%
Advanced (higher degree/A-level)	19.3%	30.6%
GCSE or equivalent	21.6%	21.2%
Other/no qualification	26.6%	24.8%
Occupational status		
Managerial/professional	23.6%	21.4%
Intermediate/small employment/own account	13.3%	12.9%
lower supervision or lower technician/semi-routine or routine	21.8%	17.5%
Not working (incl. Unemployed, retired, full-time student, people with long-term health conditions)	41.1%	48.2%
Annual personal gross income^2^		
Under £20 k	66.6%	58.1%
£20-35 k	21.2%	21.6%
£35 k+	12.2%	20.2%
Housing tenure		
House owner	64.7%	61.6%
Social renter	23.6%	23.7%
Private renter	11.7%	14.7%
*Geographical regions*		
Regional locations		
North (North East, North West and Yorkshire and the Humber)	31.3%	30.6%
Midlands (East Midlands and West Midlands)	20.5%	19.2%
South (London, South East, South West and East)	48.2%	50.2%
Settlement type		
Rural	12.3%	15.8%
Urban	87.7%	84.2%

Notes: ^1^A six-point scale, ranging from “not once in the last 12 months”, “once in the last 12 months”, “twice in the last 12 months”, “less often than once a month but at least 3 or 4 times a year”, “less often than once a week but at least once a month” to “at least once a week”. ^2^For the Taking Part survey, personal income is based on the household member with the highest income

Table 2 shows the association between neighbourhood deprivation (20% most deprived vs 20% least deprived areas) and arts engagement frequency. Of the UKHLS sample, individuals who lived in the most deprived neighbourhoods were significantly less likely to engage in all types of activities (arts participation: ATT = -0.38, 95%CI = -0.56, − 0.20; cultural attendance: ATT = -0.37, 95%CI = -0.49, − 0.25; museums and heritage sites: ATT = -0.41, 95%CI = -0.55, − 0.27). However, this was not found in the TP survey (Table 2).

**Table 2:** The association between neighbourhood deprivation (20% most deprived vs 20% least deprived) and arts engagement frequency

	Arts participation	Cultural attendance	Museums and heritage sites
*Understanding Society Wave 2 (2010/12; N = 14,782)*			
ATT	-0.38 (−0.56, − 0.20)***	-0.37 (− 0.49, − 0.25)***	-0.41 (− 0.55, − 0.27)***
Treatment N / control N / total N	7431 / 7020 / 14,451	7431 / 7020 / 14,451	7431 / 7020 / 14,451
*Taking Part survey (2010/11; N = 4575)*			
ATT	−0.08 (− 0.48, 0.32)	−0.11 (− 0.33, 0.10)	0.11 (− 0.07, 0.29)
Treatment N / control N / total N	2160 / 2281 / 4441	2160 / 2281 / 4441	2160 / 2281 / 4441

Notes: ATT stands for average treatment effect on treated. The 95%CI in parentheses were computed by bootstrapping with 100 replications. Statistical significance is denoted by asterisks: *** sig at 0.1%

When repeating the analysis on all of the 7 subcategories of IMD using data from UKHLS, lower arts participation, cultural attendance and museums and heritage engagement were found in areas of higher education, skills and training deprivation, employment deprivation, income deprivation, health deprivation and disability and crime. Reduced participation in arts activities was also found in areas of greater barriers to housing and services (Supplementary Table 2). When the main analysis was rerun using the 10% cut-off point for neighbourhood deprivation, we found that the participation rates in arts participation, cultural attendance and museums and heritage sites continued to be lower in the 10% most deprived neighbourhoods in the UKHLS sample, but again not for the TP sample (Supplementary Table 3). Finally, when comparing groups in the 20% most deprived neighbourhoods with groups in the 40% medium-deprived areas, the participation rates in all types of engagement were lower in both samples although the magnitudes of the coefficients were somewhat smaller than when comparing more distinct deprivation groups (Supplementary Table 4).

## Discussion

This study is one of the first to apply a robust PSM technique to examine how neighbourhood deprivation relates to health-promoting arts engagement. We found that people who live in the most deprived neighbourhoods were less likely to attend cultural events, participate in arts activities and visit museums and heritage sites, but this finding was only found in the UKHLS sample. Our finding is supported by previous research on the association between area deprivation and arts engagement [13, 14, 18, 28] but goes beyond these studies by using more sophisticated PSM techniques to show the relationship is independent of individual factors. Results remain even when using more stringent deprivation thresholds (the 10% IMD cut-point). When exploring subcategories of deprivation, lower participation for all types of arts engagement was found in neighbourhoods where there were lack of attainment and skills in the local population, poorer local employment levels, higher numbers of people experiencing income deprivation, a greater risk of health conditions, and a higher risk of victimisation. Poor physical and financial accessibility to housing and local services was also found to be associated with reduced arts participation.

It is notable that our main findings were found only in the UKHLS sample. It is likely that the more imprecise TP estimates are due to the relatively smaller sample size, as well as a higher proportion of missing data on the income variable. Consistent with this view, our sensitivity analyses when comparing groups in the 20% most deprived neighbourhoods with groups in the 40% medium-deprived neighbourhoods (where the analytical sample sizes had also increased), TP did find differences across all types of engagement, suggesting considerable agreement across the two studies.

Two lenses can be used to interpret our findings on the association of neighbourhood deprivation with lower arts engagements. On one hand, the “built environment” theory proposed by Gullon and Lovasi [47] suggests that urban infrastructure and community systems could causally influence people’s behaviour. In this view, neighbourhood-scale built environment characteristics including accessibility (e.g. transportation), attractiveness (e.g. green space and parks), community design features (e.g. street connectivity), public resources and services (e.g. facilities, arts venues and recreational amenities) as well as safety and stability — which deprived areas usually lack — might affect patterns of participation. There may simply be fewer cultural assets available in these spaces due to a shortage of regularly funded organisations or arts and cultural festivals. Equally, issues such as poor accessibility and neighbourhood design may affect individuals’ ability to access cultural venues such as museums and heritage sites. For instance, a report shows that reductions in local transport services, lack of local arts organisations and rurality (e.g. isolation and poor public transportation) were considered as practical barriers to participation in museums [48]. Also, poor safety may reduce the engagement of individuals in available activities (as suggested by our subgroup analyses). Even if there are facilities and infrastructures in deprived neighbourhoods, they are more likely to either suffer from a long period of neglect and underinvestment, low cultural budgets or low levels of advertising [14]. These may discourage people living in deprived areas from engaging in arts and cultural activities. However, it is important to note that deprived neighbourhoods are internally heterogeneous, which might not have been captured in our analysis. In England such areas comprise a mix of ethnically diverse inner cities, more peripheral suburban estates, parts of ‘left-behind’ coastal and provincial towns with weak economies as well as former coalfield and industrial areas still suffering from de-industrialisation. While some of these areas have very limited resources and services to support arts engagement, others may have the greatest cultural assets (e.g. cities such as London with excellent public transport and a multitude of venues) [49–51]. So the built environment alone probably cannot explain the findings presented here.

A second explanation is that our findings are driven by differences in behaviours of individuals within these areas. Whilst our analyses controlled for quantifiable and measured aspects of demographic and socio-economic status, we were unable to explore wider aspects of individuals’ personal characteristics, such as cultural norms and values or psychological capacity. It is likely that people from different backgrounds have different cultural capital, habits, tastes and preferences (e.g. the types of books they read and cultural activities they attend) [15, 16], and previous research has suggested that individuals’ childhood experience of arts engagement may predict adult engagement independent of socio-economic factors [52]. Conversely, for individuals living within areas of high deprivation, it is possible that socially-constructed norms and values and potentially lower levels of prior arts engagement may be reinforced by collective behaviours and be responsible for overall lower levels of engagement (as has also been suggested in Boardman et al.’s work on neighbourhood disadvantage and drug use [53]). In particular, social processes such as social contagion, social networks and interpersonal communications within communities (see Introduction) may play a role in affecting individuals’ behaviours and attitudes towards arts and culture, as well as their awareness of arts and cultural programmes, events, festivals and activities. This indeed has been reflected in our sensitivity analysis which shows that people living in the most deprived neighbourhoods continued to have lower engagement rates even when comparing to those living in the medium-deprived areas. This suggests that people living in deprived neighbourhoods may exhibit certain norms, values and attitudes (especially towards arts and culture) which eventually lead to the emergence of cultural divide across places with various levels of deprivation. In addition to collective cultural norms and values, people living in deprived neighbourhoods may themselves have lower psychological capacity (e.g. be unaware of the activities one could engage in) or experience psychological vulnerability (e.g. anxiety, distress, depression) [54], which in turn may lower their motivation to engage in the arts [55]. However, it is important to note that the relationship between people and the surrounding environment is mutually constitutive as cultural behaviours both shape and are simultaneously shaped by the neighbourhood environment. Nonetheless, the negative relationship between neighbourhood deprivation and arts engagement may still be attributed to the unobserved behaviours or characteristics of people who live in deprived areas, leading to a lower demand for cultural assets.

This study has a number of strengths. The implementation of PSM and the very rich representative data means that a high proportion of the propensity to living in a deprived neighbourhood was captured by observable factors (e.g. income and SES) and the risk of bias caused by unobservable heterogeneity is fairly low. The matching was also achieved to a high standard. This means that while causality cannot be unequivocally established, we nonetheless contend that the estimated relationships are credible. However, we acknowledge that unobserved heterogeneity may remain an issue; for example, we were unable to fully control for individuals’ cultural upbringings and other area-level factors. For example, lower arts engagement rates may be found in areas with higher ethnic minority populations or hard-pressed communities [13]. Relatedly, this study only considered neighbourhood deprivation as a proxy for neighbourhood effects. Future study is needed to identify the effects of other neighbourhood characteristics (such as ethnic diversity) as well as the duration of living in the neighbourhood with distinct features on arts engagement. Further, the threshold for neighbourhood deprivation (20% most deprived vs 20% least deprived) may be suboptimal, although we have also confirmed the robustness of our results using 10% as an alternative threshold.

Moreover, our study only investigated the frequency of engagement in participatory arts activities and with culture and heritage. It is likely that area deprivation may also affect the types of activities people engage in within these broad categories. For instance, people who do home-based arts activities may be less likely to be influenced by the place they live in than people who engage in activities within the community that are more likely to be affected by spatial factors (e.g. arts venues, classes/programmes, transportation). Due to the nature of the data, we were unable to differentiate between home- and community-based activities. We also need to disentangle the contributions of the neighbourhood environment and group-based characteristics on cultural behaviours. For instance, supplemental qualitative approaches may help provide deeper insights into the role of cultural capital and place-based schemes in advancing arts participation amongst deprived communities. Finally, evaluation research is required to assess the effectiveness of the current place-based schemes and to examine whether these schemes help improve the engagement rate in deprived areas.

## Conclusions

This study found that arts engagements are negatively associated with neighbourhood deprivation independent of individuals’ demographic backgrounds, socio-economic characteristics and regional locations. These results are relevant to current policy. A WHO report has shown wide-ranging benefits of the arts on health and wellbeing which are supported by more than 3000 studies across various countries [1]. The UK healthcare system has also identified community engagement as a strategy for supporting people with poor mental health [24–26]. However, our results reveal that area deprivation may act as a barrier that could prevent people from engaging in the arts, which in turn may exacerbate health inequalities. This finding is key when considering current arts and cultural schemes. It suggests that schemes that focus on increasing individual motivation and capacity to engage in cultural activities may also need to address potential structural or neighbourhood barriers (e.g. lack of regularly funded organisations, professional arts and cultural facilities, or permanent arts centres), that could additionally be affecting participation levels. Further, it highlights the importance of place-based schemes that focus on the specific characteristics and needs of deprived areas.

## Supplementary Information

**Additional file 1.**

## Data Availability

UKHLS data and Taking Part data is available from the UK Data Service. Understanding Society: Waves 1–10, 2009–2019 and Harmonised BHPS: Waves 1–18, 1991–2009 are available at https://beta.ukdataservice.ac.uk/datacatalogue/studies/study?id=6614. Understanding Society: Waves 1–10, 2009–2019: Special Licence Access, Census 2011 Lower Layer Super Output Areas are available at https://beta.ukdataservice.ac.uk/datacatalogue/studies/study?id=7248. Taking Part: the National Survey of Culture, Leisure and Sport, 2010–100; Adult and Child Data are available at https://beta.ukdataservice.ac.uk/datacatalogue/studies/study?id=6855. Index of Multiple Deprivation data can be obtained from https://www.gov.uk/government/statistics/english-indices-of-deprivation-2015.
