# Supplementary material for: Associations between neighbourhood deprivation and engagement in arts, culture and heritage: evidence from two nationally-representative samples
Source: BMC Public Health. 2021 Sep 16;21:1685. doi: 10.1186/s12889-021-11740-6 (PMC8444412; doi:10.1186/s12889-021-11740-6)
Supplement: Supplementary file 1 — Additional file 1. [file 12889_2021_11740_MOESM1_ESM.docx]

| **Supplementary Table 1: A list of arts engagement in the Understanding Society (UKHLS) and Taking Part surveys** | | |
| --- | --- | --- |
|  | UKHLS - All responses were measured on a six-point scale, ranging from “not once in the last 12 months”, “once in the last 12 months”, “twice in the last 12 months”, “less often than once a month but at least 3 or 4 times a year”, “less often than once a week but at least once a month” to “at least once a week”. | Taking Part - All responses were measured on a six-point scale, ranging from “not once in the last 12 months”, “once in the last 12 months”, “twice in the last 12 months”, “less often than once a month but at least 3 or 4 times a year”, “less often than once a week but at least once a month” to “at least once a week”. |
| Arts activities | - Dance, including ballet - Sang to an audience or rehearsed for a performance (not karaoke) - Played a musical instrument - Written music - Rehearsed or performed in a play/drama, opera/operetta or musical theatre - Taken part in a carnival or street arts event (as a musician, dancer or costume maker) - Learned or practised circus skills - Photography, film, or video making as an artistic activity (not family or holidays) - Used a computer to create original artworks or animation - Textile crafts, wood crafts or any other crafts, such as embroidery, knitting, wood turning furniture making, pottery or jewellery - Read for pleasure (not newspapers, magazines, or comics) - Written any stories, plays or poetry - Been a member of a book club, where people meet up to discuss and share books - Painting, drawing, printmaking or sculpture | - Ballet - Other dance (for fitness) - Other dance (not for fitness) - Sang to an audience or rehearsed for a performance (not karaoke) - Played a musical instrument to an audience or rehearsed for a performance - Played a musical instrument for your own pleasure - Written music - Rehearsed or performed in a play/drama - Rehearsed or performed in an opera/operetta or musical theatre - Taken part in a carnival (e.g. as a musician, dancer or costume maker) - Taken part in street arts (an artistic performance that takes place in everyday surroundings like parks, streets or shopping centres) - Learned or practised circus skills - Painting, drawing, printmaking or sculpture - Photography as an artistic activity (not family or holiday ‘snaps’) - Made films or videos as an artistic activity (not family or holidays) - Used a computer to create original artworks or animation - Textile crafts such as embroidery, crocheting or knitting - Wood crafts such as wood turning, carving or furniture making - Other crafts such as calligraphy, pottery or jewellery for yourself - Written any stories or plays - Written any poetry - Reading as part of a group |
| Cultural events | - A film at a cinema or other venue - Exhibition or collection or art, photography or sculpture or a craft exhibition (not craft market) - Event which included video or electronic art - Event connected with books or writing - Street arts or a public art display or installation (art in everyday surroundings, or an art work such as sculpture that is outdoors or in a public place) - Carnival or culturally specific festival (for example, Mela, Baisakhi, Navrati, Feis) - Circus (not animals) - Play/drama, pantomime or a musical - Opera/operetta - Classical music performance - Rock, pop or jazz performance - Ballet - Contemporary dance - African people’s dance or south Asian and Chinese dance | - Film at a cinema or other venue - Exhibition or collection of art, photography or sculpture - Craft exhibition (not crafts market) - Event which included video or electronic art - Event connected with books or writing - Street arts (art in everyday surroundings like parks, streets or shopping centres) - A public art display or installation (an art work such as sculpture that is outdoors or in a public place) - Circus (not animals) - Carnival - Culturally specific festival (for example, Mela, Baisakhi, Navrati) - Play/drama - Pantomime - Musical - Opera/operetta - Classical music performance - Jazz performance - Other live music event - Ballet - Contemporary dance - African people’s dance or South Asian and Chinese dance - Other live dance event |
| Museums and heritage sites | - Visit a museum or gallery - Visit any of the following heritage sites: - A city or town with historic character - A historic building open to the public (non-religious) - A historic park or garden open to the public - A place connected with industrial history (e.g. an old factory, dockyard or mine) or historic transport system (e.g. an old ship or railway) - A historic place of worship attended as a visitor (not to worship) - A monument such as a castle, fort or ruin - A site of archaeological interest (e.g. Roman villa, ancient burial site) - A site connected with sports heritage (e.g. Wimbledon) (not visited for the purposes of watching sport) | - Visit a museum or gallery - Visit any of the following heritage sites: - A city or town with historic character - A historic building open to the public (non-religious) - A historic park or garden open to the public - A place connected with industrial history (e.g. an old factory, dockyard or mine) or historic transport system (e.g. an old ship or railway) - A historic place of worship attended as a visitor (not to worship) - A monument such as a castle, fort or ruin - A site of archaeological interest (e.g. Roman villa, ancient burial site) - A site connected with sports heritage (e.g. Wimbledon) (not visited for the purposes of watching sport) |


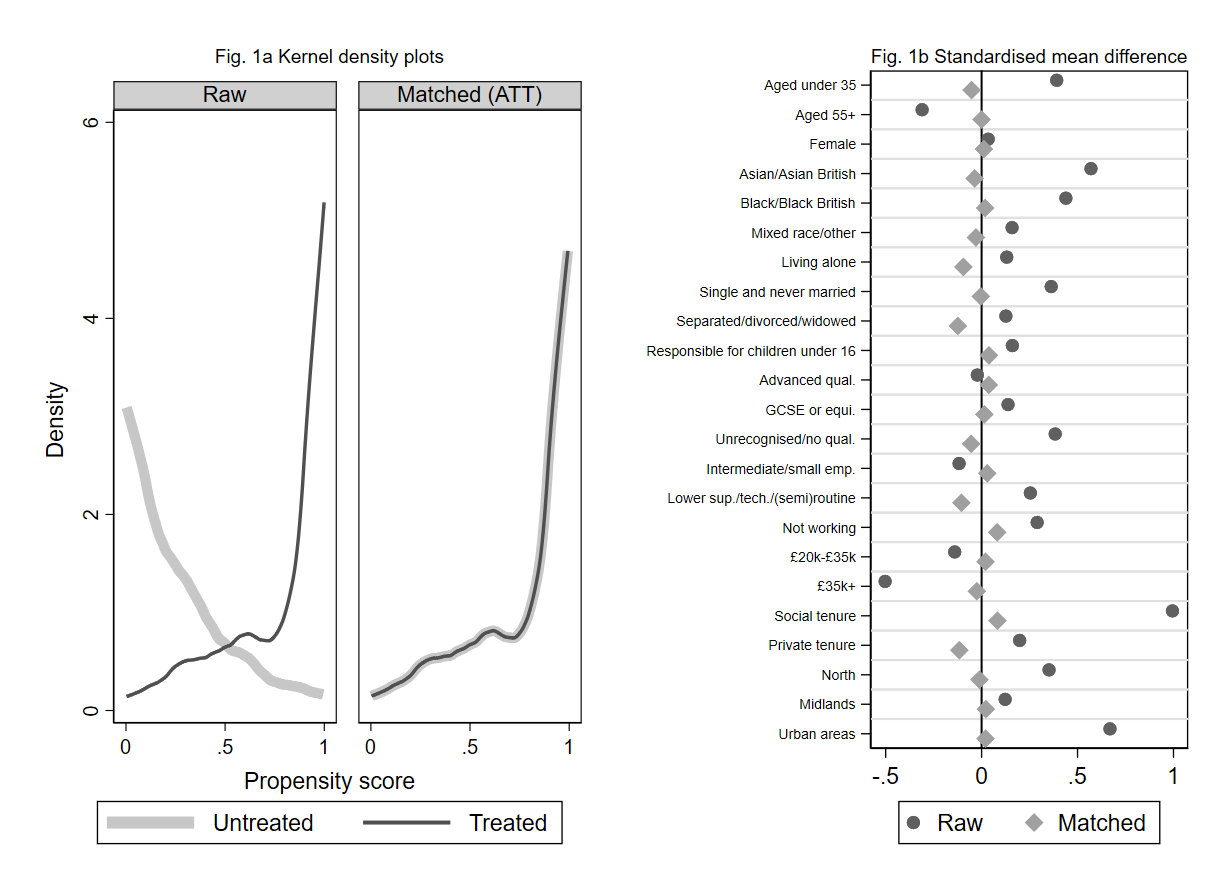


Supplementary Figure s1b Standardised mean difference

Supplementary Figure s1a Kernel density plots

**Data source: Understanding Society Wave 2 (2010/12).**

**Supplementary Figure s1a: Kernel density distributions of the observed variables between the treated and untreated before (raw) and after matching (matched). Supplementary Figure s1b: Standardised mean difference of the covariates for the treated and untreated before and after matching.**


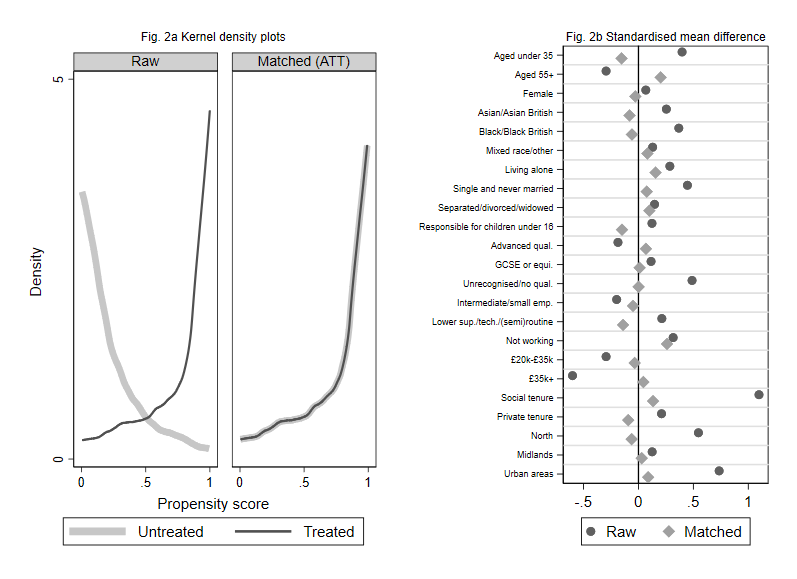


Supplementary Figure s2b Standardised mean difference

Supplementary Figure s2a Kernel density plots

**Data source: Taking Part Survey (2010/11).**

**Supplementary Figure s2a: Kernel density distributions of the observed variables between the treated and untreated before (raw) and after matching (matched). Supplementary Figure s2b: Standardised mean difference of the covariates for the treated and untreated before and after matching.**

| **Supplementary Table 2: The 7 subcategories of the IMD domain (20% most deprived vs 20% least deprived) using the Understanding Society Wave 2 (2010/12) survey** | | | |
| --- | --- | --- | --- |
|  | **Arts participation** | **Cultural attendance** | **Museums and heritage sites** |
| *Education, skills and training deprivation^1^ (N=14,144)* | | |  |
| ATT | -0.34 (-0.50, -0.18)*** | -0.40 (-0.50, -0.31)*** | -0.49 (-0.59, -0.40)*** |
| Treatment N / control N / total N | 7,085 / 6,910 / 13,995 | 7,085 / 6,910 / 13,995 | 7,085 / 6,910 / 13,995 |
| *Employment deprivation^2^ (N=14,180)* | |  |  |
| ATT | -0.45 (-0.62, -0.29)*** | -0.38 (-0.52, -0.23)*** | -0.38 (-0.51, -0.24)*** |
| Treatment N / control N / total N | 7,382 / 6,766 / 14,148 | 7,382 / 6,766 / 14,148 | 7,382 / 6,766 / 14,148 |
| *Income deprivation^3^ (N=14,547)* | |  |  |
| ATT | -0.51 (-0.77, -0.26)*** | -0.33 (-0.48, -0.17)*** | -0.43 (-0.59, -0.26)*** |
| Treatment N / control N / total N | 7,716 / 6,557 / 14,273 | 7,716 / 6,557 / 14,273 | 7,716 / 6,557 / 14,273 |
| *Health deprivation and disability^4^ (N=14,161)* | |  |  |
| ATT | -0.49 (-0.70, -0.29)*** | -0.42 (-0.58, -0.26)*** | -0.32 (-0.48, -0.15)*** |
| Treatment N / control N / total N | 7,010 / 6,849 / 13,859 | 7,010 / 6,849 / 13,859 | 7,010 / 6,849 / 13,859 |
| *Crime^5^ (N=14,970)* |  |  |  |
| ATT | -0.42 (-0.59, -0.26)*** | -0.29 (-0.43, -0.15)*** | -0.26 (-0.37, -0.14)*** |
| Treatment N / control N / total N | 7,390 / 7,300 / 14,690 | 7,390 / 7,300 / 14,690 | 7,390 / 7,300 / 14,690 |
| *Barriers to housing and services^6^ (N=15,065)* | |  |  |
| ATT | -0.26 (-0.44, -0.08)** | -0.05 (-0.23, 0.12) | -0.08 (-0.22, 0.05) |
| Treatment N / control N / total N | 8,087 / 6,940 / 15,027 | 8,087 / 6,940 / 15,027 | 8,087 / 6,940 / 15,027 |
| *Living environment deprivation^7^ (N=14,536)* | |  |  |
| ATT | -0.10 (-0.24, 0.03) | -0.05 (-0.14, 0.03) | -0.01 (-0.09, 0.08) |
| Treatment N / control N / total N | 7,439 / 7,094 / 14,533 | 7,439 / 7,094 / 14,533 | 7,439 / 7,094 / 14,533 |
| Notes: ATT stands for average treatment effect on treated. The 95%CI in parentheses were computed by bootstrapping with 100 replications. Statistical significance is denoted by asterisks: ** sig at 1%, *** sig at 0.1%.  ^1^ Education, skills and training deprivation measures the lack of attainment and skills in the local population. ^2^ Employment deprivation measures the proportion of the working age population who were involuntarily excluded from the labour market. ^3^ Income deprivation measures the proportion of population experiencing deprivation relating to low income. ^4^ Health deprivation and disability measures the risk of premature death and the impairment of quality of life through poor physical or mental health. ^5^ Crime measures the risk of victimisation at local level. ^6^ Barriers to housing and services measures the physical and financial accessibility of housing and local services. ^7^ Living environment deprivation measures the quality of “indoor” and “outdoor” local environment. More information on IMD can be found here: Department for Communities and Local Government. The English Indices of Deprivation 2015, Sept 2015, online available at: https://assets.publishing.service.gov.uk/government/uploads/system/uploads/attachment_data/file/465791/English_Indices_of_Deprivation_2015_-_Statistical_Release.pdf. | | | |

| **Supplementary Table 3: The association between neighbourhood deprivation (10% most deprived vs 10% least deprived) and arts engagement frequency** | | | |
| --- | --- | --- | --- |
|  | **Arts participation** | **Cultural attendance** | **Museums and heritage sites** |
| *Understanding Society Wave 2 (2010/12; N=7,286)* | | |  |
| ATT | -0.65 (-1.01, -0.29)*** | -0.36 (-0.56, -0.15)** | -0.48 (-0.71, -0.25)*** |
| Treatment N / control N / total N | 3,469/ 3,256 / 6,725 | 3,469/ 3,256 / 6,725 | 3,469/ 3,256 / 6,725 |
| *Taking Part survey (2010/11; N=2,304)* | |  |  |
| ATT | -0.21 (-0.75, 0.33) | -0.23 (-0.50, 0.05) | -0.07 (-0.35, 0.21) |
| Treatment N / control N / total N | 804 / 1,069 / 1,873 | 804 / 1,069 / 1,873 | 804 / 1,069 / 1,873 |
| Notes: ATT stands for average treatment effect on treated. The 95%CI in parentheses were computed by bootstrapping with 100 replications. Statistical significance is denoted by asterisks: ** sig at 1%, *** sig at 0.1%. | | | |

| **Supplementary Table 4: The association between neighbourhood deprivation (20% most deprived vs 40% medium levels of deprivation) and arts engagement frequency** | | | |
| --- | --- | --- | --- |
|  | **Arts participation** | **Cultural attendance** | **Museums and heritage sites** |
| *Understanding Society Wave 2 (2010/12; N=22,249)* | | |  |
| ATT | -0.21 (-0.31, -0.11)*** | -0.17 (-0.24, -0.10)*** | -0.18 (-0.25, -0.11)*** |
| Treatment N / control N / total N | 7,706 / 14,471 / 22,177 | 7,706 / 14,471 / 22,177 | 7,706 / 14,471 / 22,177 |
| *Taking Part survey (2010/11; N=6,845)* | |  |  |
| ATT | -0.20 (-0.35, -0.05)* | -0.14 (-0.23, -0.05)** | -0.17 (-0.27, -0.08)*** |
| Treatment N / control N / total N | 2,254 / 4,546 / 6,800 | 2,254 / 4,546 / 6,800 | 2,254 / 4,546 / 6,800 |
| Notes: ATT stands for average treatment effect on treated. The 95%CI in parentheses were computed by bootstrapping with 100 replications. Statistical significance is denoted by asterisks: * sig at 5%, ** sig at 1%, *** sig at 0.1%. | | | |
